# Role of CgTpo4 in Polyamine and Antimicrobial Peptide Resistance: Determining Virulence in *Candida glabrata*

**DOI:** 10.3390/ijms22031376

**Published:** 2021-01-29

**Authors:** Mafalda Cavalheiro, Daniela Romão, Rui Santos, Dalila Mil-Homens, Pedro Pais, Catarina Costa, Mónica Galocha, Diana Pereira, Azusa Takahashi-Nakaguchi, Hiroji Chibana, Arsénio M. Fialho, Miguel C. Teixeira

**Affiliations:** 1 Department of Bioengineering, Instituto Superior Técnico, Universidade de Lisboa, 1049-001 Lisbon, Portugal; mafalda.cavalheiro@tecnico.ulisboa.pt (M.C.); daniela.romao@irbbarcelona.org (D.R.); rui.ramossantos@uzh.ch (R.S.); dalilamil-homens@tecnico.ulisboa.pt (D.M.-H.); pedrohpais@tecnico.ulisboa.pt (P.P.); catarinaspcosta@ist.utl.pt (C.C.); monicagalocha@tecnico.ulisboa.pt (M.G.); diana.pereira@tecnico.ulisboa.pt (D.P.); afialho@tecnico.ulisboa.pt (A.M.F.); 2 Biological Sciences Research Group, IBB—Institute for Bioengineering and Biosciences, Instituto Superior Técnico, 1049-001 Lisbon, Portugal; 3 Medical Mycology Research Center (MMRC), Chiba University, Chiba 263-8522, Japan; azusan_takahashi@faculty.chiba-u.jp (A.T.-N.); chibana@faculty.chiba-u.jp (H.C.)

**Keywords:** *Candida glabrata*, CgTpo4, virulence, *Galleria mellonella*, AMP resistance, polyamine resistance

## Abstract

*Candida glabrata* is an emerging fungal pathogen whose success depends on its ability to resist antifungal drugs but also to thrive against host defenses. In this study, the predicted multidrug transporter CgTpo4 (encoded by ORF *CAGL0L10912g*) is described as a new determinant of virulence in *C. glabrata*, using the infection model *Galleria mellonella*. The *CgTPO4* gene was found to be required for the *C. glabrata* ability to kill *G. mellonella*. The transporter encoded by this gene is also necessary for antimicrobial peptide (AMP) resistance, specifically against histatin-5. Interestingly, *G. mellonella’s* AMP expression was found to be strongly activated in response to *C. glabrata* infection, suggesting AMPs are a key antifungal defense. CgTpo4 was also found to be a plasma membrane exporter of polyamines, especially spermidine, suggesting that CgTpo4 is able to export polyamines and AMPs, thus conferring resistance to both stress agents. Altogether, this study presents the polyamine exporter CgTpo4 as a determinant of *C. glabrata* virulence, which acts by protecting the yeast cells from the overexpression of AMPs, deployed as a host defense mechanism.

## 1. Introduction

*Candida* species are among the top 10 most frequently isolated nosocomial bloodstream pathogens, with an annual incidence rate of up to 4.8 cases in Europe and 13.3 cases in the US per 100,000 inhabitants [1]. Despite the fact that *Candida albicans* is still the predominant cause of invasive candidiasis, a higher proportion of patients has been infected by non-*albicans Candida* species [2]. *Candida glabrata* has risen as the most frequently non-*albicans* pathogenic *Candida* species isolated from candidemia patients, accounting for 20% of the diagnosed cases of candidiasis in both Europe and North America [3,4]. The frequency and relative high mortality levels (up to 45% for *C. glabrata*) of these infections [5] are generally attributed to the capacity of these pathogenic yeasts to efficiently develop multidrug resistance (MDR), tolerate host defense mechanisms, maintain high proliferative and repopulation capacity through biofilm formation, and to display the ability to withstand prolonged harmful conditions such as nutrient starvation and oxidative stress [6,7].

*C. glabrata* can resist the confrontation with host immune cells, as a significant fraction of phagocytised cells are able to survive and replicate inside the macrophages. Since the host defense mechanisms are multifaceted, a number of different evasion strategies have evolved [8]. These include avoidance of contact with macrophages, rapid escape from host immune cells, ability to withstand macrophage antimicrobial activities and, most importantly, use of macrophages as an intracellular niche for protection and replication processes [8]. In addition to this, in the event of *Candida* infection, other virulence factors, such as biofilm development and resistance to antimicrobial peptides, also play crucial roles for colonization and infection establishment [9]. Antimicrobial peptides, small positively charged amphipathic molecules, work directly against microbes, mainly through a mechanism that involves membrane disruption and pore formation, meaning that being able to resist them can be highly positive for the yeast to complete its infection cycle [10].

During the past three decades, antifungal drug resistance became a serious concern for the medical community. The fact that *C. glabrata* isolates are able to quickly develop resistance to new drugs enables them to keep causing prevalent infectious that are difficult to eliminate [11]. Transporters of the Drug:H^+^ antiporter (DHA) family, belonging to the Major Facilitator Superfamily (MFS), well characterized in the yeast model *Saccharomyces cerevisiae*, are known to take part in MDR as well as in other important physiological stress response mechanisms, including weak acid and polyamine resistance and transport [12,13,14].

In this work, the predicted *C. glabrata* DHA CgTpo4, encoded by the ORF *CAGL0L10912g*, is shown to play a role in virulence against the infection model *G. mellonella*. ORF *CAGL0L10912g* was predicted to encode an ortholog of the *S. cerevisiae TPO4* gene, which was characterized as a determinant of resistance to polyamines [15]. Within the DHA family in *C. glabrata*, eight other homologous transporters have been characterized so far [14]. These include CgAqr1, CgDtr1, CgFlr1, CgFlr2, CgQdr2, CgTpo1_1, CgTpo1_2 and CgTpo3, which were found to confer resistance to drugs and other stress factors, such as azoles, flucytosine, benomyl, mancozeb, acetic acid and polyamines, mostly by contributing to decrease the intracellular accumulation of those molecules [13,16,17,18,19,20]. Additionally, the *C. albicans* DHA transporters CaMdr1, CaNag3, CaNag4, CaQdr1, CaQdr2 and CaQdr3, as well as the *C. glabrata* DHA transporters CgTpo1_1, CgTpo1_2 and CgDtr1, were found to further play a role in these yeasts’ virulence, although the exact molecular mechanisms behind this observation are still to be fully elucidated [20,21,22,23,24,25].

Here, the impact of *CgTPO4* expression in the context of virulence against the infection model *G. mellonella* was addressed. The larval immune system has high similarity to the mammalian innate immune system in both their phagocytic cells and the humoral responses, involving antimicrobial peptides, to eliminate infecting microbes [26]. The influence of CgTpo4 in the success of *C. glabrata*’s infection in this host and the consequent impacts on the larval survival rate were evaluated. Given the obtained indications, the role of CgTpo4 in this process was investigated, with emphasis on its activity in antimicrobial peptide resistance. These elements allow a deeper understanding of the mechanisms involved in *C. glabrata* virulence, a poorly characterized subject.

## 2. Results

### 2.1. CgTPO4 Is a Determinant of C. glabrata Virulence against the G. mellonella Infection Model

**Figure 1:**
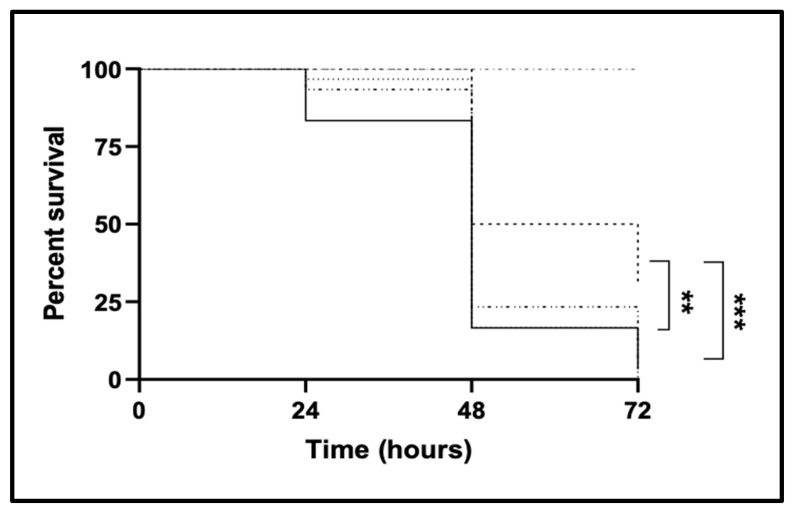
*CgTPO4* expression is required for *C. glabrata* virulence against the *G. mellonella* infection model. The survival of larvae injected with approximately 5 × 10^6^ CFU/larvae of wild-type KUE100 *C. glabrata*, harboring the pGREG576 cloning vector ( ), or the pGREG576_MTI_*CgTPO4* expression plasmid ( ), and of *C. glabrata* deletion mutant *Δcgtpo4*, harboring the pGREG576 cloning vector ( ) or the pGREG576_MTI_*CgTPO4* expression plasmid ( ), with PBS for control ( ) are displayed as Kaplan–Meier survival curves. The displayed results are the average of at least three independent experiments, with standard deviation represented by the grey lines. ** *p <* 0.01, *** *p <* 0.001.

Using *G. mellonella* larvae as an infection model, the possible effect of CgTpo4 transporter in *C. glabrata* ability to exert its virulence was assessed, resorting to the overexpression of its encoding gene in the wild type of *C. glabrata*, and to its deletion in the parental strain, followed by its complementation. The survival of the larvae was evaluated after 24, 48 and 72 h upon injection of 5 × 10^6^ of fungal cells. The results show that deleting the *CgTPO4* gene in the wild-type background reduces its virulence in this infection model. In fact, after 72 h the wild type leads to the survival of only 20% of the initial larvae population, while 50% of the larvae are able to survive when infected with *Δcgtpo4* deletion mutant. Reinserting the *CgTPO4* gene in the deletion mutant *Δcgtpo4* has shown to lead to a decrease in the survival of *G. mellonella* larvae, to levels very similar to the ones the *C. glabrata* wild-type strain provokes. Meanwhile, overexpressing the *CgTPO4* gene in the wild-type background did not increase the larvae killing (Figure 1).

### 2.2. CgTPO4 Confers Resistance to the Human Antimicrobial Peptide Histatin-5, But Not to Phagocytosis

Based on the observation that *CgTPO4* increases *C. glabrata* ability to kill *G. mellonella* larvae, an effort was put into the identification of the role of CgTpo4 in this context.

The first hypothesis tested was that CgTpo4 could protect the yeast cells from the stress induced during phagocytosis. To assess this, *C. glabrata* cells were exposed to a cell culture of *G. mellonella* hemocytes. The concentration of viable *C. glabrata* cells found within the hemocytes was measured after 1, 4, 24 and 48 h after coculture. At all time-points, the number of viable wild-type cells internalized within hemocytes was found to be undistinguishable from the number of *Δcgtpo4* internalized cells (results not shown).

**Figure 2:**
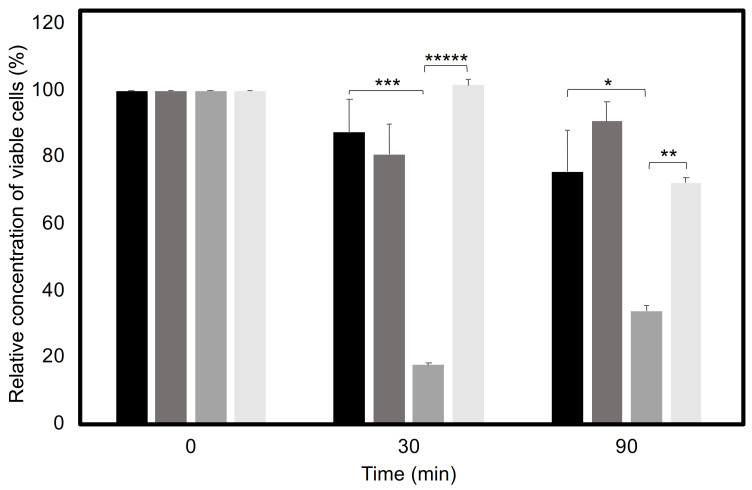
CgTpo4 is a determinant of histatin-5 resistance. Relative concentration of viable cells of wild-type *C. glabrata* KUE100, harboring the pGREG576 cloning vector (black), or the pGREG576_MTI_*CgTPO4* expression plasmid (dark grey) and the derived *∆cgtpo4* deletion mutant, harboring the cloning vector pGREG576 (grey), or the expression plasmid pGREG576_MTI_*CgTPO4* (light grey), in the presence of 35 µM of histatin-5. The average percentage of survival, obtained from at least three independent experiments, is indicated by the black lines, with standard deviation being represented by error bars. * *p <* 0.05, ** *p <* 0.01, *** *p <* 0.001, and ***** *p <* 0.00001.

Another possible mechanism by which the CgTpo4 transporter may increase *C. glabrata*’s ability to kill *G. mellonella* larvae is by mediating resistance to antimicrobial peptides (AMPs) present in the insect’s hemolymph. To test if this transporter is involved in AMP resistance, cell cultures of both wild-type and derived *∆cgtpo4* deletion mutants were grown until mid-exponential phase and incubated in the presence of the human AMP histatin-5, which is known to possess candidacidal properties [27]. Upon 1 h 30 min of incubation in the presence of 35 μM histatin-5, about the same concentration found in the human saliva, approximately 80% of the *C. glabrata* wild-type population was seen to survive, whereas only 30% of the *∆cgtpo4* mutant population remains viable in the same period. Furthermore, the overexpression of *CgTPO4* in the wild-type background increases the survival of *C. glabrata* to almost 100%, while the complementation of *∆cgtpo4* deletion mutant with the *CgTPO4* gene recovers the survival of *C. glabrata* cells to wild-type levels (Figure 2). These results might explain why the deletion of this transporter causes yeast cells to become less virulent, since the increased effectiveness of the action of antimicrobial peptides on the mutant cells might be compromising the progress of infection.

### 2.3. Antimicrobial Peptide Gene Expression Is Highly Activated in G. mellonella Larvae in Response to Infection by C. glabrata

**Figure 3:**
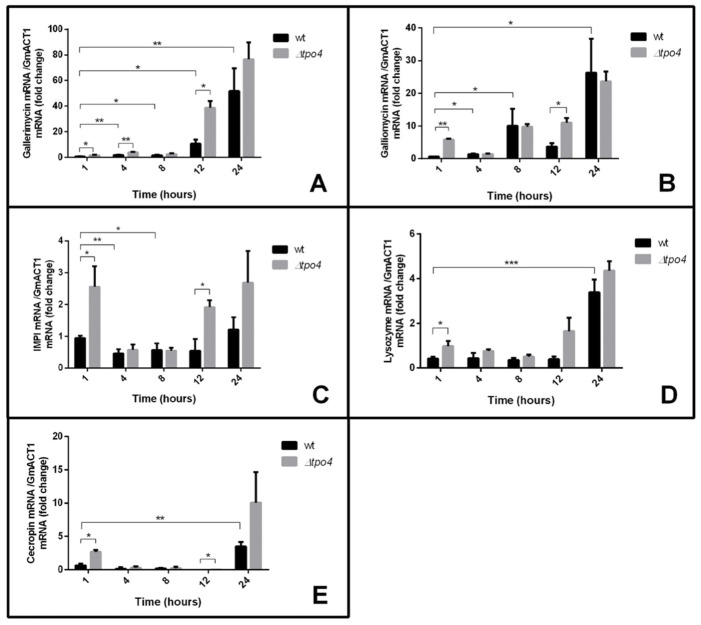
Expression of genes encoding antimicrobial peptide (AMPs) is activated in *G. mellonella*, during infection by *C. glabrata*. Comparison of the variation of the gallerimycin (**A**), galliomycin (**B**), inducible metalloproteinase inhibitor (IMPI) (**C**), lysozyme (**D**) and cecropin (**E**) transcript levels in *G. mellonella*, after 1, 4, 8, 12, and 24 h of infection by KUE100 *C. glabrata* wild-type cells (black bars) and derived *Δcgtpo4* mutant cells (grey bars). The presented transcript levels were obtained by quantitative RT-PCR and are normalized to the *GmACT1* mRNA levels, relative to the values registered in *G. mellonella* larvae, in the same time points, treated with PBS buffer as a control. The indicated values are averages of at least three independent experiments. Error bars represent the corresponding standard deviations. * *p <* 0.05; ** *p <* 0.01; *** *p <* 0.001.

In order to evaluate the importance of AMPs in the response to *C. glabrata* infection, the transcript levels of five immune-responsive genes, encoding peptides with antimicrobial properties, were assessed through quantitative real-time PCR (RT-PCR). The assessed AMPs were gallerimycin, galliomycin, inducible metalloproteinase inhibitor (IMPI), lysozyme and cecropin. Interestingly, a stepwise increase in the expression of gallerimycin and galliomycin in larvae injected with *C. glabrata* cells was observed—this increase reached dramatic 60-fold and 25-fold higher transcript levels at 24 h after infection when compared to control larvae, respectively (Figure 3A,B). The results obtained for the other three tested AMPs were less impressive, but still significant with lysozyme and cecropin encoding genes showing a four-fold upregulation after 24 h of *C. glabrata* infection (Figure 3D,E), suggesting that these AMPs constitute a later response to fight the fungal infection. No significant change in IMPI expression during infection by *C. glabrata* was registered (Figure 3C).

To evaluate whether the decreased virulence exhibited by *∆cgtpo4* deletion mutant, when compared to the wild-type strain, could be related to decreased ability of *G. mellonella* to detect the mutated pathogen and activate its immune response, the expression of the same AMP encoding genes was measured in *G. mellonella* larvae exposed to *∆cgtpo4* mutant cells. Interestingly, the deletion of *CgTPO4* did not significantly change the larval response to infection in terms of AMP expression, except for the levels registered after 1 and 12 h of exposure, where the larvae appear to respond even more intensely to the mutant strain, when compared to the wild-type strain (Figure 3A–E).

Altogether, these results show that *C. glabrata* cells are recognized as external agents stimulating the host immune responses in the infection model *G. mellonella*. Additionally, CgTpo4 does not seem to affect the host’s ability to activate its defenses.

### 2.4. CgTPO4 Is a Determinant of Polyamine Resistance in C. glabrata

Given that the TPO transport proteins have been characterized as polyamine transporters in *S. cerevisiae* [15,28] and in *C. glabrata* [13,17], it seemed relevant to evaluate if CgTpo4 had similar transport abilities. This effect appeared particularly relevant since AMP transport has been linked to polyamine transporters, as exemplified in the case of histatin-5 in *C. albicans* cells [29].

**Figure 4:**
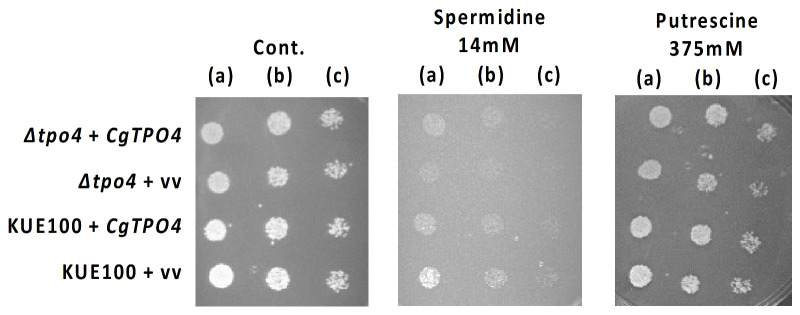
*CgTPO4* confers resistance to polyamines in *C. glabrata*. Comparison of the susceptibility to the polyamines, spermidine and putrescine, at the indicated concentrations, of the *C. glabrata* KUE100 wild-type strain harboring the pGREG576 cloning vector (KUE100 + vv) or the pGREG576_MTI_*CgTPO4* vector (KUE100 + *CgTPO4*) and the derived KUE100_*Δcgtpo4* mutant cells harboring the pGREG576 cloning vector (*Δcgtpo4* + vv) or the pGREG576_MTI_*CgTPO4* vector (*Δcgtpo4* + *CgTPO4*), in YPD agar plates, by spot assays. The inocula were prepared as described in the Materials and Methods section. Cell suspensions used to prepare the spots were 1:5 (**b**) and 1:25 (**c**) dilutions of the cell suspension used in (**a**). The displayed images are representative of at least three independent experiments.

To this end, the susceptibility against inhibitory concentrations of polyamines exhibited by *C. glabrata* wild-type cells, overexpressing the *CgTPO4* gene or not, as well as by *Δcgtpo4* deletion mutant cells and *Δcgtpo4* deletion mutant cells complemented with *CgTPO4* gene, was evaluated through spot assays (Figure 4). *Δcgtpo4* mutant cells showed higher spermidine and putrescine susceptibility than the wild-type cells, a stronger effect being apparent for spermidine. While the overexpression of *CgTPO4* was found not to significantly increase the resistance to spermidine and putrescine, the complementation of *∆cgtpo4* deletion mutant with *CgTPO4* gene restored almost completely the resistance observed in the wild-type strain to these two polyamines. Using a similar approach, no role in the resistance to antifungal drugs of the azole, polyene or fluoropyrimidine families was identified for *CgTPO4* (results not shown).

### 2.5. CgTpo4 Is a Plasma Membrane Polyamine Exporter

**Figure 5:**
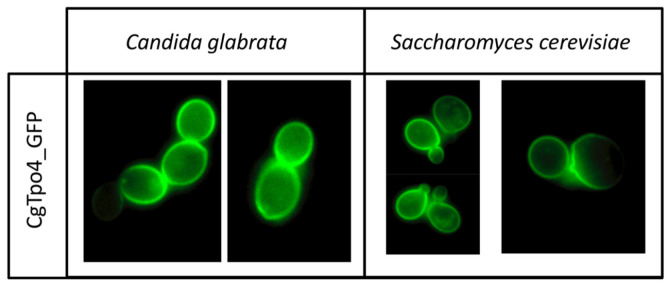
CgTpo4 is localized in the plasma membrane of yeast cells. Fluorescence of exponential-phase L5U1 *C. glabrata* cells or BY4741 *S. cerevisiae* cells, harboring the pGREG576_MTI_*CgTPO4* or pGREG576_*CgTPO4* plasmids, after 5 h of copper- or galactose-induced recombinant protein production, respectively. Results indicate that the CgTpo4_GFP fusion protein localizes to the plasma membrane in both *S. cerevisiae* and *C. glabrata* cells.

In order to assess the role of CgTpo4 in polyamine resistance, it is crucial to confirm its subcellular localization. In *C. glabrata* cells harboring the pGREG576_MTI_*CgTPO4* plasmid, the CgTpo4_GFP fusion protein was found to be predominantly localized to the cell periphery (Figure 5). *S. cerevisiae* cells harboring the pGREG576_*CgTPO4* plasmid were also tested for the subcellular localization of CgTpo4, to verify if this transporter was similarly localized (Figure 5).

**Figure 6:**
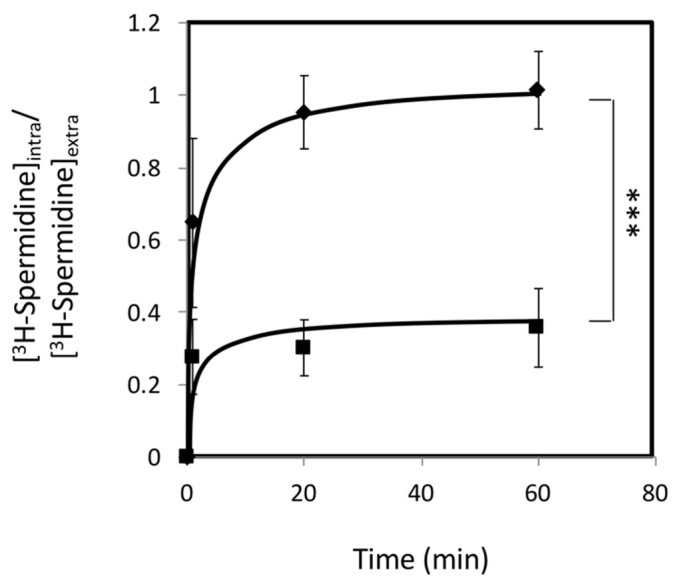
*CgTPO4* expression leads to decreased spermidine accumulation in *C. glabrata*. Time course accumulation ratio of [^3^H]-Spermidine in nonadapted cells of KUE100 *C. glabrata* wild-type (■) or KUE100_*Δcgtpo4* (♦) strains, during cultivation in YPD liquid medium in the presence of 8.5 mM unlabeled spermidine. The accumulation ratio values are averages of at least three independent experiments. Error bars represent the corresponding standard deviations. *** *p <* 0.001.

To assess the exact role of this transporter in polyamine resistance, the intracellular accumulation of radiolabeled [^3^H]-spermidine was assessed for both wild-type and *Δcgtpo4* cells. The *Δcgtpo4* deletion mutant exhibited three-fold higher intracellular accumulation of radiolabeled spermidine when compared with the wild-type strain, meaning that *CgTPO4* expression is strongly related to a decreased polyamine accumulation in *C. glabrata* cells (Figure 6). Therefore, and in resemblance to its orthologue in *S. cerevisiae*, there are strong indications to suggest that the CgTpo4 transporter plays a role in polyamine resistance, by mediating the extrusion of polyamines from within the yeast cell to the extracellular medium.

## 3. Discussion

In this study, the CgTpo4 transporter, predicted to be a multidrug transporter of the MFS, is demonstrated to play a role in *C. glabrata* virulence, this effect being proposed to be based on its ability to confer AMP resistance.

CgTpo4 was shown to be required for the ability of *C. glabrata* cells to kill the infection model *G. mellonella*. Since this model system presents an immune system which is highly similar to the mammalian innate immune system, dependent on phagocytic cells (hemocytes and macrophages, respectively) that allow *C. glabrata* to survive and replicate for a prolonged time, and the release of antimicrobial stress agents such as AMPs, the use of *Galleria mellonella* is considered a suitable model to understand virulence mechanisms in human hosts [24,30,31]. In fact, *G. mellonella* is widely used for testing virulence of fungi and antifungal efficacy of new compounds, being considered a suitable infection model to study *Candida, Cryptococcus*, *Aspergillus* and other fungal species, while decreasing costs, time and ethical considerations in comparison to mammalian models [32].

During the triggering of an inflammatory response, neutrophils and macrophages are recruited and basophils and mast cells in the nearby connective tissues are stimulated to produce peptides that help eliminating the foreign pathogen [33]. Increased levels of AMP expression in *G. mellonella* had been observed upon exposure to *Salmonella enterica* serovar *typhimurium*, reaching after 16 h of injection 80-fold, 10-fold and 15-fold increases in gallerimycin, galliomycin and lysozyme expression, respectively [34]. In the case of infection by *Legionella pneumophila*, *G. mellonella* larvae displayed three-fold higher expression of gallerimycin and galliomycin after 24 h of injection [35]. In this study, the activation of immune-response genes encoding antimicrobial peptides in response to *C. glabrata* were observed for the first time, demonstrating that these mechanisms are indeed activated in *G. mellonella* against *C. glabrata* infections. Interestingly, antifungal activity had been registered in *G. mellonella* hemolymph [36]. Additionally, some of the *G. mellonella* AMPs have been shown to display antifungal activity, specifically lysozyme has been shown to inhibit *C. albicans* growth [37], while gallerimycin was found to exhibit activity against the environmental fungi *Matarhizium anisophiae* [38], *Sclerotinia minor* and *Erysiphe cichoracearum* [39]. The profile of AMP activation induced by *C. glabrata* is slightly different from that registered during infections with other species, suggesting that *G. mellonella* perception and reaction to the presence of pathogens varies from species to species. In the case of *C. glabrata*, registered herein, gallerimycin and galliomycin seem to be the most activated AMPs, eventually corresponding to the most effective AMPs against this fungal pathogen in *G. mellonella*.

In the attempt to find the molecular basis of CgTpo4 activity as a virulence determinant, CgTpo4 was found not to affect *C. glabrata* proliferation upon phagocytosis, but rather to contribute to the resistance to AMPs. Indeed, CgTpo4 was found to confer resistance to the human AMP histatin-5. Histatins are histidine-rich antimicrobial proteins found in saliva secreted by Ebner’s glands, and they offer early defense against incoming microorganisms, especially fungi [40]. Histatin-5 is the smallest of the major salivary histatins and the most active in terms of its antifungal properties that resorts to proteolysis to eliminate threats [40]. The observation made in this study that CgTpo4 confers histatin-5 resistance is coherent with the previous observation that two other members of the DHA family—CaFlu1 in *C. albicans* [41] and CgTpo1_1, in *C. glabrata* [24]—also confer resistance to this AMP. These facts point to the possibility that several members of the DHA family, including CgTpo4, are required for full protection against AMPs, in both larval and human hosts, contributing in this fashion to increase yeast survival and proliferation during the infection cycle.

Given that the TPO transport proteins have been characterized as polyamine exporters in *S. cerevisiae* [15,28] and in *C. glabrata* [13,17], and the fact that AMP transport has been linked to polyamine transporters [29], it seemed relevant to evaluate if CgTpo4 had similar polyamine transport functions. Although no role in antifungal drug resistance could be attributed to CgTpo4, this transporter was found to act as a plasma membrane polyamine exporter. Since *Δcgtpo4* deletion mutant cells were shown to display on average a two-fold higher plasma membrane potential than wild-type cells [42], it is possible to suggest that this transporter is involved, directly or indirectly, in the maintenance of plasma membrane potential, by controlling the homeostasis of polycationic polyamines and entrance of H^+^ inside the cell. This appears to be consistent with the participation of several DHA transporters in the transport of cations and polycations in *S. cerevisiae* [15,43,44] and in *C. glabrata* [13,17], a process that is bound to affect plasma membrane potential. Indeed, CgTpo4 was previously implicated in the control of the plasma membrane potential, thus affecting biofilm formation in *C. glabrata* [42]. Given the importance of CgTpo4 in biofilm formation and AMP resistance, it would be interesting to assess if biofilms are involved in the resistance to histatin-5, or if the action of CgTpo4 on the plasma membrane potential affects these two processes independently.

The role of CgTpo4 is even more necessary upon the presence of both spermidine and histatin-5 simultaneously. Polyamines are present in the saliva of healthy individuals and increases in concentration in pancreatic cancer patients [45]. Given the presence of both molecules in the saliva is important to consider the effects of histatin-5 together with other components of the saliva.

Previous reports have already shown that DHA encoding genes *CaQDR1*, *CaQDR2* and *CaQDR3*, in *C. albicans* [22], and *CgDTR1*, *CgTPO1_1* and *CgTPO1_2*, in *C. glabrata* [20,24], are involved in the infection outcome of pathogenic yeasts. This study adds CgTpo4 to this battery of virulence factors coming from a family of transporters of drugs, xenobiotics and inhibitory chemical compounds, reinforcing the notion that effectors of resistance to host-induced stress are key factors in pathogenesis, and constitute interesting new drug targets for antifungal therapy.

## 4. Materials and Methods

### 4.1. Strains, Plasmids and Growth Medium

*Candida glabrata* CBS138, KUE100 [46], KUE100::URA- [47], *∆cgtpo4* [42] and L5U1 (cgura3∆0 cgleu2∆0) [16] strains, the latter kindly provided by John Bennett, NIAID, NIH, Bethesda, were used in this study. *C. glabrata* cells were cultivated in YPD medium, containing: 20 g/L D-(+)- glucose (Merck, Darmstadt, Germany), 20 g/L bacterial-peptone (LioChem, Conyers, GA, USA) and 10 g/L of yeast extract (Difco, BD, England, United Kingdom). BM basal minimal medium used for the susceptibility assays contained 20 g/L glucose (Merck, Darmstadt, Germany), 1.7 g/L yeast nitrogen base without amino acids or NH_4_+ (Difco BD, England, United Kingdom) and 2.65 g/L (NH_4_)_2_SO_4_ (Merck, Darmstadt, Germany). *S. cerevisiae* BY4741 wild-type strain, used in gene cloning through homologous recombination, was grown in either YPD medium or MM4-U medium, which resulted from BM supplementation with 20 mg/L methionine, 20 mg/L histidine and 60 mg/L leucine (all from Merck, Darmstadt, Germany). *C. glabrata* L5U1 strains were cultured in BM medium supplemented with 20 mg/L uracil and 60 mg/L leucine. *C. glabrata* KUE100::URA- and KUE100_*Δcgtpo4*:URA- strains, the latter constructed in this study, harboring pGREG576 derived plasmids were grown in BM.

### 4.2. Cloning of the C. glabrata CgTPO4 Gene (ORF CAGL0L10912g)

The pGREG576 plasmid from the Drag and Drop collection [48] was used to clone and express the *C. glabrata* ORF *CAGL0L10912g* in *S. cerevisiae*, as described before for other heterologous genes [49]. *CAGL0L10912g* DNA was generated by PCR, using genomic DNA extracted from the sequenced CBS138 *C. glabrata* strain, and the primers indicated in Table 1. The amplified fragment was cotransformed into *S. cerevisiae* BY4741 strain with the pGREG576 vector, digested with SalI, to obtain through homologous recombination the pGREG576_*CgTPO4* plasmid. To enable *CgTPO4* expression in *C. glabrata*, the *GAL1* promoter was replaced by the copper-inducible MTI *C. glabrata* promoter, giving rise to the pGREG576_MTI_*CgTPO4* plasmid. The MTI promoter DNA was generated by PCR, using genomic DNA extracted from the CBS138 *C. glabrata* strain, and the primers indicated in Table 1. The amplified fragment was cotransformed into the *S. cerevisiae* BY4741 strain with the pGREG576_*CgTPO4* plasmid, digested with SacI and NotI to remove the *GAL1* promoter, to generate through homologous recombination the pGREG576_MTI_*CgTPO4* plasmid. Constructs were verified by DNA sequencing.

### 4.3. Disruption of C. glabrata CgURA3 (ORF CAGL0I03080g)

The disruption of the *C. glabrata URA3* gene encoded by ORF CAGL0I03080g, was carried out in the KUE100_*Δcgtpo4* mutant, using the CRISPR-Cas9 system from Vyas et al. [50]. Briefly, a *CgURA3* gRNA sequence selected from the resources made available by Vyas et al. [50] was cloned in the pV1382 plasmid, previously linearized with the restriction enzyme BsmBI (NEB). The *CgURA3* gRNA was obtained by oligonucleotide annealing and the product ligated into the previously linearized pV1382 plasmid to obtain the pV1382_*CgURA3* vector. The construct was verified by DNA sequencing. The plasmid was transformed into the KUE100_*Δcgtpo4* strain and cells were then directly plated on 5-Fluoroorotic acid (5-FOA) to select for URA- cells. Sequential passages in nonselective medium (YPD) were performed to avoid detrimental effects of further Cas9 expression and *CgURA3* loss of function was further confirmed by the inability to grow in medium without uracil. The introduction of pGREG576 derived plasmids in the edited strains was able to rescue the growth impairment in the absence of uracil.

### 4.4. Galleria Mellonella Survival Assays

*Galleria mellonella* larvae were reared in our lab insectarium, on a pollen grain and bee wax diet at 25 °C in the darkness. Last instance larvae weighting 250 ± 25 mg were used in the survival assays and the larvae infection was based on the protocol previously described [51]. *C. glabrata* strains were cultivated in BM medium and transferred to YPD containing 150 µmM CuSO_4_, and growth until stationary phase was achieved. Cells were then harvested by centrifugation and resuspended in PBS (pH 7.4). A micrometer device was used to control a microsyringe and inject 5 µL of yeast cell suspension (approximately 5 × 10^6^ cells per injection) into each caterpillar via the last left proleg, which had been previously surface sanitized with 70% (*v/v*) ethanol. Following injection, larvae were placed in Petri dishes and stored in the dark at 37 °C. For each condition, 10 larvae were used to follow the larval survival over a period of 72 h. Caterpillars were considered dead when they displayed no movement in response to touch. Control larvae were injected with PBS (pH 7.4).

### 4.5. Galleria Mellonella Hemocyte–Yeast Interaction Assays

To isolate *G. mellonella* hemocytes, larvae previously anesthetized on ice and surface sterilized with 70% (*v/v*) ethanol were punctured in the abdomen with a sterile needle and the outflowing hemolymph was immediately collected into a sterile microtube containing anticoagulant buffer (98 mM NaOH, 145 mM NaCl, 17 mM EDTA, 41 mM citric acid (pH 4.5)) in a 1:1 proportion. The hemolymph was centrifuged at 250× *g* for 10 min at 4 °C to pellet hemocytes and washed twice with PBS (centrifuged at 250× *g* for 5 min at 4 °C). The hemocyte pellet was then gently suspended in 1 mL of Grace insect medium (GIM) (Sigma) supplemented with 10% fetal bovine serum, 1% glutamine and 1% antibiotic (10,000 U penicillin G, 10 mg streptomycin). Suspended hemocytes were counted with a hemocytometer and incubated at 25 °C in 24-well plates at a concentration of 2 × 10^5^ cells/mL. Monolayers of primary *Galleria* hemocytes were used for experiments the next day. All preparations and assays were carried out under sterile conditions.

Cultures of *C. glabrata* cells were grown in YPD medium until mid-exponential phase (OD_600nm_ = 0.4–0.6). The optical density of the cultures was measured, and the appropriate volume was collected to have 7 × 10^2^ cells/mL in PBS. *Galleria* hemocyte monolayer medium was replaced with GIM without antimycotics, and then cells were infected with the yeast suspensions with a multiplicity of infection (MOI) of 1:5. After 1 h of infection at 37 °C, the hemocytes were carefully washed twice with PBS, followed by the addition of GIM. The viable intracellular yeast cells were quantified 1, 4, 24 and 48 h after infection. Cell monolayers were lysed with 0.5% Triton X-100, and Colony Forming Units (CFUs) were determined by plating dilutions of cell lysates on YPD-agar plates followed by incubation at 30 °C for 24 h.

### 4.6. Gene Expression Measurement

The transcript levels of genes encoding the *G. mellonela* antimicrobial peptides gallerimycin, galliomycin, IMPI, lysozyme and cecropin were determined by RT-PCR. Three larvae per treatment for each time point (10 and 21 h postinfection) were cryopreserved, sliced and homogenized in 1 mL of Trizol reagent (Merck, Darmstadt, Germany). Whole animal RNA was extracted according to the manufacturer’s protocol. RNA was treated with Turbo DNase (Ambion, Applied Biosystems, Thermo Fisher Scientific, MA, USA) according to manufacturer’s recommendations and quantified spectrophotometrically (NanoDrop ND-1000, Thermo Fisher Scientific, MA, USA). Total RNA was converted to cDNA for the real-time Reverse Transcription PCR using the MultiScribe Reverse Transcriptase kit (Applied Biosystems, Thermo Fisher Scientific, MA, USA) and the 7500 RT-PCR thermal cycler block (Applied Biosystems, Thermo Fisher Scientific, MA, USA). The real-time PCR step was carried out using adequate primers (Table 1), SYBR Green^®^ reagents (Applied Biosystems, Thermo Fisher Scientific, MA, USA) and the 7500 RT-PCR thermocycler block (Applied Biosystems, Thermo Fisher Scientific, MA, USA). Default parameters set by the manufacturer were followed, and fluorescence was detected by the instrument and plotted in an amplification graph (7500 Systems SDS Software, Applied Biosystems, Thermo Fisher Scientific, MA, USA). *G. mellonella* Actin encoding gene transcript level was used as an internal reference.

### 4.7. Candidacidal Assays of Histatin-5

To evaluate the effect of *CgTPO4* expression in histatin-5 resistance, *C. glabrata* cells were batch cultured in minimal medium, containing 150 µmM CuSO_4_ until mid-exponential phase (OD_600nm_ = 0.4–0.6)_._ Cells were then washed twice and resuspended in sterile PBS to an OD_600nm_ = 0.01 ± 0.001 and supplemented with 35 µM of histatin-5 (Sigma). Cell suspensions were incubated at 30°C under agitation (250 rpm) and cell viability was measured at 30 min intervals, based on the counting of CFU, determined by plating dilutions of the cell suspensions on YPD-agar plates followed by incubation at 30 °C for 48 h. Each result was compared with the initial cell content of the suspension in order to obtain a survival curve.

### 4.8. Subcellular Localization of the CgTpo4 Transporter

*C. glabrata* L5U1 cells harboring the pGREG576_MTI_*CgTPO4* plasmid were grown to mid-exponential phase in minimal medium containing 150 µmM CuSO_4_. At a standard OD_600nm_ of 0.5 ± 0.05, obtained after around 5h of incubation, cells were inspected through fluorescence microscopy. *S. cerevisiae* BY4741 cells harboring the pGREG576_*CgTPO4* plasmid were grown in MM4-U medium with 1% galactose to induce protein expression. The distribution of CgTpo4_GFP protein in *S. cerevisiae* or in *C. glabrata* living cells was detected by fluorescence microscopy in a Zeiss Axioplan microscope (Carl Zeiss MicroImaging, Oberkochen, Germany), using excitation and emission wavelengths of 395 and 509 nm, respectively. Fluorescence images were captured using a cooled CCD camera (Cool SNAPFX, Roper Scientific Photometrics, Tucso, AZ, USA).

### 4.9. Susceptibility Assays in C. glabrata

The susceptibility of *C. glabrata* cells towards toxic concentrations of the selected drugs and polyamines was evaluated as before through spot assays [18,49]. *C. glabrata* cell suspensions used to inoculate the agar plates were mid-exponential cells grown in adequate minimal medium containing 150 µmM CuSO_4_, until culture OD_600nm_ = 0.6 ± 0.02 was reached and then diluted in sterile water to obtain suspensions with OD_600nm_ = 0.05 ± 0.005. These cell suspensions and subsequent dilutions (1:5; 1:25) were applied as 4 µL spots onto the surface of solid medium containing 150 µmM CuSO_4_, without uracil supplemented with adequate chemical stress concentrations. The tested drugs and chemicals included the following compounds, used in the specified concentration ranges: the polyamines spermidine (4–14 mM) and putrescine (300–400 mM), the azole antifungal drugs fluconazole (10–200 mg/L), ketoconazole (10–50 mg/L), clotrimazole (1–20 mg/L), tioconazole (0.2–1 mg/L) and miconazole (0.2–1 mg/L), the polyene antifungal drug amphotericin B (0.1–0.5 mg/L) and the fluoropyrimidine 5-flucytosine (0.02–5 mg/L).

### 4.10. Spermidine Accumulation Assays

To analyze the accumulation ratio (intracellular/extracellular concentration) of radiolabeled [^3^H]-spermidine (Amersham Biosciences, Buckinghamshire United Kingdom) in *C. glabrata* wild-type and *∆cgtpo4* strains, the two were grown in YPD medium (at pH 4.0) until the mid-exponential phase (OD_600nm_ = 0.5 ± 0.05), harvested by filtration, washed with fresh medium and finally resuspended in 5 mL of this same medium to obtain dense cell suspensions (OD_600nm_ = 0.7 ± 0.05). These cell suspensions were incubated for 5 min at 30 °C with agitation (150 rpm). After that time, 0.1 µM of [^3^H]-spermidine (Amersham Biosciences, Buckinghamshire United Kingdom) was added to the cell suspension together with 8.5 mM unlabeled spermidine (Merck, Darmstadt, Germany). The intracellular accumulation of radiolabeled spermidine was followed during 60 min by filtering, at adequate intervals, 200 µL of the cell suspension through prewetted glass microfiber filters (Whatman GF/C). The filters were washed with ice-cold Tris-Magnesium Sulfate buffer (0.1 M Mes, 41 mM Tris, both from Sigma, adjusted to pH 4.0) and the radioactivity was measured in a Beckman LS 5000TD scintillation counter. Extracellular concentration of [^3^H]-spermidine was estimated by measuring the radioactivity of 50 µL of the culture supernatant. Intracellular concentration of [^3^H]-spermidine was calculated considering the internal cell volume (Vi) of the two strains constant and equal to 2.5 mL (mg dry weight)^−1^ [52].

### 4.11. Statistical Analysis

Statistical analysis of the G. mellonella survival assay results was performed using the Mantel–Cox test. For the remaining results, statistical analysis was performed using Graphpad Prism Software version 6.0 (La Jolla, CA, USA) and analyzed with Student*’*s *t-*test. *p*-values equal or inferior to 0.05 were considered statistically significant. Error calculations were made considering an n ≥ 30 in the G. mellonella survival assays and *n* ≥ 3 in the remaining cases.

## Figures and Tables

**Table 1:** List of primers used in this study. Unless otherwise indicated, the primers were generated in this study.

Name	Sequence (5′-3′)
*CgTPO4* gene cloning	
pGREG_CgTPO4 _Fw	GAATTCGATATCAAGCTTATCGATACCGTCGACAATGGCCGGTACAAATCAAG
pGREG_CgTPO4_Rev	GCGTGACATAACTAATTACATGACTCGAGGTCGACCTATACCATTCTAGAGGAG
pGREG576 GAL-to-MTI promotor replacement	
pGREG_MTI_ Fw	TTAACCCTCACTAAAGGGAACAAAAGCTGGAGCTCTGTACGACACGCATCATGTGGCAATC
pGREG_MTI_Rev	GAAAAGTTCTTCTCCTTTACTCATACTAGTGCGGCTGTGTTTGTTTTTGTATGTGTTTGTTG
*CgURA3* gene disruption	
*CgURA3* gRNA in pV1382 _Fw	GATCGACCGGCCAAGGTATCGTCACG
*CgURA3* gRNA in pV1382 _Rev	AAAACGTGACGATACCTTGGCCGGTC
RT-PCR experiments	
P1RT Galle	CGCAATATCATTGGCCTTCT [53]
P2RT Galle	CCTGCAGTTAGCAATGCAC [53]
P1RT Galli	TCGTATCGTCACCGCAAAATG [54]
P2RT Galli	GCCGCAATGACCACCTTTATA [54]
P1RT IMPI	AGATGGCTATGCAAGGGATG [53]
P2RT IMPI	AGGACCTGTGCAGCATTTCT [53]
P1RT Lys	TCCCAACTCTTGACCGACGA [53]
P2RT Lys	AGTGGTTGCGCCATCCATAC [53]
P1RT Cecr	ATTTGCCTGCATCGTAGCG [55]
P2RT Cecr	CTTGTACTGCTGGACCAGCTTTT [55]
P1RT Act	ATCCTCACCCTGAAGTACCC [53]
P2RT Act	CCACACGCAGCTCATTGTA [53]

## Data Availability

Data is contained within the article.
